# Translation, cultural adaptation, and content validation of the Hong Kong Chinese version of Self-completion Adult Social Care Outcomes Toolkit (ASCOT-SCT4) for care service users

**DOI:** 10.1186/s12955-025-02389-5

**Published:** 2025-06-04

**Authors:** Siyue Yu, Judy Chu Dik Sze, Annie Wai Ling Cheung, Kailu Wang, Elizabeth Welch, Nick Smith, Richard Huan Xu, Eliza Lai Yi Wong

**Affiliations:** 1 JC School of Public Health and Primary Care, Faculty of Medicine, The Chinese University of Hong Kong, Hong Kong SAR, China; 2 Center for Health Systems and Policy Research, JC School of Public Health and Primary Care, Faculty of Medicine, The Chinese University of Hong Kong, Hong Kong SAR, China; 3 Personal Social Services Research Unit, University of Kent, Canterbury, UK; 4 Centre for Health Service Studies, University of Kent, Canterbury, UK; 5 Department of Rehabilitation Sciences, The Hong Kong Polytechnic University, Hong Kong SAR, China

**Keywords:** ASCOT, Long-term care, Quality of life, Cross-cultural adaption, Cognitive interviews, Content validity, Outcome assessment

## Abstract

**Background:**

In light of the global challenges posed by an ageing population, the evaluation of long-term care (LTC) is of particular importance. The Adult Social Care Outcomes Toolkit Four-Level Self-completion Tool (ASCOT-SCT4) is a preference-based instrument developed to measure long-term care (LTC) related quality of life (QoL). However, it is not yet available in Hong Kong (HK). This study aims to translate and culturally adapt the ASCOT-SCT4 into Chinese and evaluate its content validity in the HK context.

**Methods:**

The study adhered to well-established international guidelines for conducting translation, cultural adaptation, and content validation of instruments. The translation process included forward, backward translations, and expert committee review. Subsequently, LTC users aged 60 or above and experts with diverse professional backgrounds in LTC were involved in content validation in terms of comprehensibility, relevance, and comprehensiveness through cognitive interviews and a content validity index (CVI) survey. For clarity and relevance, an item-level CVI (I-CVI) of ≥ 0.78 and a scale-level CVI (S-CVI) of ≥ 0.90 were considered acceptable.

**Results:**

In the translation process, concern was raised about the literal translation of ‘*Control over daily life*’ item. During the cognitive interviews, 27 LTC users perceived the instrument’s length as acceptable, and experienced no sensitive feelings while completing it. However, seven of the nine items were found to have comprehension and interpretation issues, which were mostly resolved through revisions of wordings. For the ‘*Food and drink*’ and ‘*Dignity*’ items, alternative terms were used and sentence structure was revised to improve its comprehensibility while ensuring equivalence to the original English version. The final translated version demonstrated acceptable clarity (S-CVI: 0.92, I-CVIs: 0.86–1.00) as evaluated by seven experts. Both LTC users and experts found the items relevant (S-CVI: 0.97, I-CVIs: 0.94 to 1.00), and no additional LTC-related QoL domains were identified as missing from the instrument.

**Conclusions:**

This study provides evidence that the Chinese version of the ASCOT-SCT4 is comprehensible, relevant, and comprehensive for the HK context, which allows for further testing on psychometric properties in a larger population.

**Supplementary Information:**

The online version contains supplementary material available at 10.1186/s12955-025-02389-5.

## Background

With the global trend of an ageing population [1], the demand for long-term care (LTC) is rapidly increasing, imposing significant burdens on health and social care systems and presenting notable societal challenges [2, 3]. Consequently, the development of affordable and sustainable LTC systems is increasingly recognized as crucial across nations [4, 5]. Quality of life (QoL) is a critical self-reported outcome for evaluating the quality and (cost-)effectiveness of care services, aiding in improvement action and better resource allocation [6–8]. To gather QoL data, a valid and reliable instrument is essential [9]. Over the past decades, health-related instruments such as the EuroQol-5 Dimension (EQ-5D) [10] have been extensively used to measure outcomes in health and social care services [11–13]. However, these instruments, which focus primarily on health, may inadequately capture the impacts of LTC, whose main objective is not to cure diseases but to compensate for the loss of independent functioning, support independent living, and ultimately enhance well-being [14, 15].

In response to this concern, the Adult Social Care Outcomes Toolkit (ASCOT), a preference-based instrument, was developed in 2010 by a research team in the United Kingdom (UK) to measure the impact and value of LTC, also known as social care in the UK [16, 17]. The instrument comprises nine items covering eight domains, which include both basic aspects (*Personal cleanliness, Food and drink, Personal safety, and Home cleanliness*) and higher-order aspects of living (*Control over daily life, Social participation, Occupation, and Dignity*), with each item having four response options (ideal state, no needs, some needs, and high needs). The ASCOT is a suite of instruments (https://www.pssru.ac.uk/ascot/), of which the self-completion version (SCT4) is the most commonly used. The ASCOT-SCT4 has demonstrated validity and reliability in various countries [18–22], and is recommended by the National Institute for Health and Care Excellence for assessment and economic evaluation of social care [23]. Still, to apply the ASCOT-SCT4 in other locations/regions, systematic translation, cultural adaptation, and subsequent validation of the instrument are necessary to ensure the instrument is appropriate, acceptable, comprehensible, culturally relevant, valid, and reliable in local contexts [24, 25].

To our knowledge, no such instrument is available for assessing the (cost-)effectiveness of LTC in Hong Kong (HK), a city on the path to becoming a ‘super-aged society’ where 20% of the population was aged 65 or above in 2021, with this figure projected to rise to 34% by 2046 [26]. This demographic shift has driven the HK government to significantly increase public spending on LTC services in recent years [27]. Despite this, the needs of LTC remain unmet: as of Oct 2023, the waiting time for subsidized residential care homes has reached 22 months [28], and for home care services, it has reached 7 months [29]. This highlights the urgent need to develop or translate a culturally appropriate instrument to facilitate policymakers in efficiently allocating resources from a cost-effective perspective in the LTC sector. By adapting an existing validated instrument, we can take advantage of previous research, avoid redundant development efforts, and, more importantly, facilitate cross-cultural data comparisons [24, 30]. The ASCOT-SCT4 may serve as a promising instrument; however, there is a lack of evidence regarding its measurement properties in HK. Poor translation and cultural adaptation may lead the translated version to diverge in meanings and concepts from the original source, threatening the validity of data collected and compromising the aggregation of global data sets [31]. Furthermore, inadequate content validity may cause response errors, leading to unreliable data that hinders the investigation of relationships between the outcome and the other variables, as well as the actual changes in participants’ status [32]. Therefore, it is crucial to ensure the equivalence between the translated version and the original source. Moreover, the content validity should be regarded as a fundamental prerequisite for selecting an appropriate instrument and ensuring its further psychometric properties [32, 33].

As the first and the most important phase towards developing a culturally validated instrument, this study aimed to: (1) translate the ASCOT-SCT4 into Traditional Chinese and undertake a cross-cultural adaptation to ensure semantic equivalence (the words in the translated instrument hold equivalent meaning to the original) and conceptual equivalence (the domains in the translated instrument reflect the same concepts as the original) [34]; and (2) examine the content validity in terms of comprehensibility (whether the respondents understand the instrument as intended), relevance (whether the items are relevant to the concept being measured), and comprehensiveness (whether the instrument covers all important aspects of the concept) of the adapted version for the HK context [32]. The goals of the present study were to yield a HK Chinese version of the ASCOT-SCT4 that reaches equivalence to the original English version for assessing QoL in LTC locally and internationally, and to support international data comparisons.

## Methods

### Design of the study

**Fig. 1:**
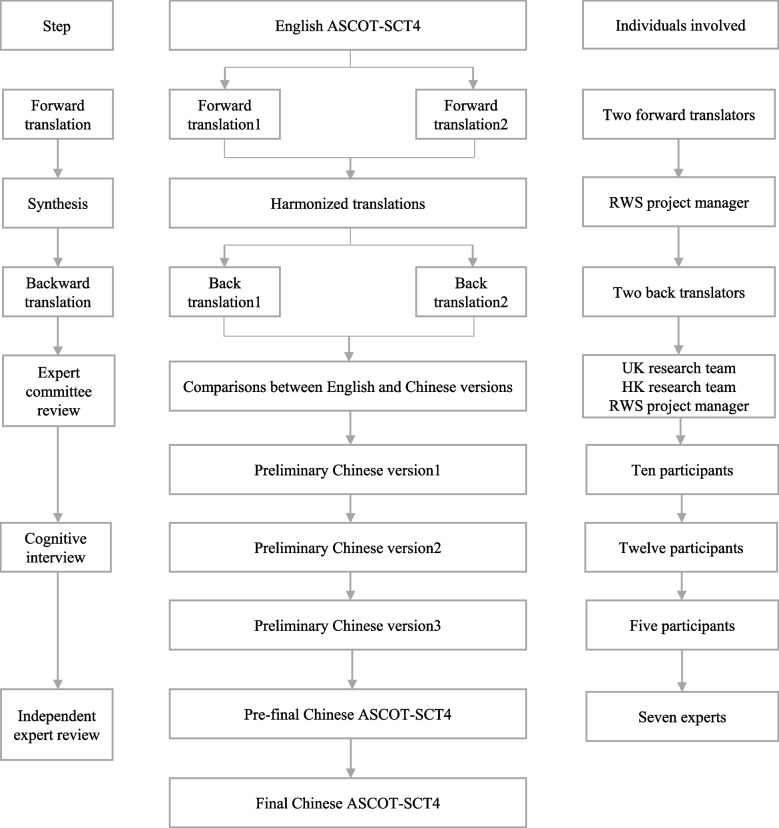
Process of translation, cross-cultural adaptation, and content validation of Chinese ASCOT-SCT4

This study adhered to two well-established international guidelines: the ISPOR guideline for good practices in translation and cultural adaptation for patient-reported outcome measures [31], and the COnsensus-based Standards for the selection of health Measurement Instruments (COSMIN) methodology for assessing content validity [35]. The translation process was carried out by our research team (HK research team) in collaboration with the original developers from the UK (UK research team) and the professional translation company RWS Life Sciences (RWS). The process of translation, cross-cultural adaptation, and content validation of the instrument is shown in Fig. 1. The study is reported following COSMIN reporting guideline for studies of measurement properties (Table S1) [36].

#### Step 1: Forward translation

Two bilingual translators (F1 & F2), native Chinese speakers with high English fluency and experience translating patient-reported outcome questionnaires, independently translated the ASCOT-SCT4 from English into Chinese. The translators were provided with the instrument’s concept elaboration guide (Table S2).

#### Step 2: Harmonized synthesis

A project manager from RWS resolved discrepancies between the two translations (F1 & F2) and harmonized them into a single Chinese version. The HK research team then reviewed this harmonized version and provided feedback to ensure it was comprehensible and the wording appropriate for the HK context. The project manager from RWS then revised the translation based on this feedback.

#### Step 3: Back translation

Two other bilingual translators (B1 & B2), native English speakers with high Chinese fluency, were blinded to the original English version and back-translated the ASCOT-SCT4 from Chinese into English. All the translators (F1, F2, B1, and B2) were employed by RWS.

#### Step 4: Expert committee review

The translation reports, including the harmonized synthesis and back translations, were reviewed by the UK research team, the HK research team, and the project manager from RWS. Discrepancies and concerns regarding semantic and conceptual equivalence to the English source version were discussed and resolved by the committee until a consensus was reached. Eventually, a preliminary version of the Chinese ASCOT-SCT4 was agreed upon and made ready for cognitive debriefing interviews.

#### Step 5: Cognitive interviewing

##### Participants and recruitment

Participants were eligible if they: (1) were aged 60 or over, (2) were LTC service users, (3) could speak Cantonese and read Chinese, and (4) had no cognitive impairment that would prevent participation (e.g., dementia). Participants were excluded if they had difficulty reading the questionnaire or were too weak to complete the interview. Purposive sampling was employed to recruit participants with diverse characteristics regarding age, gender, education, living conditions, and types of LTC services used. Recruitment strategies included active approaches with eligibility screenings through collaborating NGOs, and passive approaches including website advertisement and leaflet distribution. As recommended by previous qualitative studies, a sample of around 20–30 participants was deemed sufficient to achieve data saturation [37, 38]. The cognitive interviewing process underwent three rounds of iterative refinement and continued until the Chinese ASCOT-SCT4 demonstrated good comprehension while retaining equivalence to the source. Written informed consent was obtained from all participants.

##### Interview procedure

The cognitive interviews, conducted by two trained interviewers, utilized think-aloud and verbal probing techniques with a semi-structured interview guideline (Supplementary file) [39]. The primary objective was to assess participants’ comprehension of the instrument’s items and response options. Participants were asked to interpret each of the items and the corresponding response options in their own words, provide examples to illustrate their understanding, explain their choice of responses, and give feedback on any difficulties encountered. The secondary objective was to evaluate the instrument’s relevance and comprehensiveness. Participants were asked about the relevance of the items to their QoL and whether any important aspects of their QoL were not included in the instrument. Cross-cultural appropriateness was also assessed by asking if participants experienced any sensitive or uncomfortable feelings while completing the questionnaire.

Interviews were conducted one-on-one and face-to-face. Due to the COVID-19 pandemic, phone interviews were also conducted for participants unable to attend in person. At the start of each interview, the interviewers explained the study’s aim and the cognitive interviewing procedure. Participants were then instructed to complete the questionnaire using the think-aloud method. Following this, the interviewers asked participants for their initial impressions of the instrument’s clarity, ease of comprehension, and acceptability of its length, and probed them about each item. Participants were then asked to rate the relevance of each item using a 4-point Likert scale, with 1 indicating “not relevant” and 4 indicating “very relevant”. At the end of the interviews, participants were asked about whether they thought any important domains related to their QoL and that could be delivered or influenced by LTC were missing from the instrument. Each interview lasted between 30 and 60 min, and participants were given a HKD100 supermarket voucher as a token of appreciation for their contribution to the study. Both face-to-face and phone interviews followed the same interview procedure and guideline. While participants in face-to-face interviews reviewed the instrument physically, those in phone interviews had the items and response options read aloud to them. To avoid introducing potential bias, interviewers were well-trained to read the instrument verbatim without adding interpretation or commentary.

#### Step 6: Independent expert review

This step is not included in the ISPOR guideline [31], but it was considered important to evaluate the relevance and comprehensiveness from experts’ perspectives, as recommended by the COSMIN guideline [35], and to gather feedback on the experts’ judgements regarding clarity in order to finalize the Chinese ASCOT-SCT4. Previous research suggested involving between 5 and 10 experts in the content validation process [40]. The experts were emailed a content validation form to rate each item’s relevance and clarity on a Likert scale from 1 (not relevant/clear) to 4 (very relevant/clear) and to provide comments and suggestions for improvement. Additionally, they were asked to indicate any key domains related to LTC that should be added, and to identify any existing domains that are redundant. Then, the HK research team prepared a summary report and discussed it with the UK research team to confirm the final version of the Chinese ASCOT-SCT4.

### Data analysis

All interviews were audio-recorded and transcribed verbatim. The cognitive interview data regarding comprehensibility were analyzed using a coding framework developed by a Dutch research team based on Tourangeau’s model of the question-response process [41]. This framework has been employed in exploring the content validity of the Dutch ASCOT-SCT4 [42], and adapted to categorize the findings related to comprehensibility (Table S3). Summarized qualitative findings and proposed revisions were discussed with the UK research team and RWS. Quantitative ratings on clarity (by experts) and relevance (by experts and participants) were dichotomized: ratings of 1 and 2 were categorized as ‘not relevant/not clear,’ and ratings of 3 and 4 were categorized as ‘relevant/clear [43]. The Item-Content Validity Index (I-CVI) for relevance/clarity was calculated by dividing the number of experts/participants rating items as clear/relevant (rating 3 or 4) by the total number of experts/participants. An I-CVI of at least 0.78 was considered acceptable [40]. The scale CVI (S-CVI) was calculated using the average (S-CVI/Ave) method by dividing the sum of I-CVIs by the total number of items. An S-CVI/Ave of ≥ 0.90 indicated satisfactory content validity [40]. Findings on comprehensiveness were summarized qualitatively based on feedback from participants and experts.

## Result

### Translation (Steps 1–4)

Concern was raised about one item during the translation process. The literal translation of ‘control’ in ‘*Q1. Control over daily life*’ item could have negative connotations and was sometimes associated with machine control or authoritative behaviour in the HK context. Therefore, the term was replaced with ‘autonomy’ for further testing.

### Cross-cultural adaptation and content validation (Step 5–6): cognitive interviews and independent expert review

#### Participant and expert characteristics

The cognitive interviewing process was performed in three rounds with 27 participants between November 2022 and April 2023. Table 1 presents the sample characteristics of participants from the cognitive interviews. The independent expert review was conducted between April and May 2023, during which seven experts with professional backgrounds in LTC were invited. Table 2 presents their demographic characteristics.

**Table 1:** Characteristics of the participants from cognitive interview (*N* = 27)

Variables	Group	Total *N* = 27 (%)	Round 1 *n* = 10 (%)	Round 2 *n* = 12 (%)	Round 3 *N* = 5 (%)
Age range	60–69	11 (41%)	5 (50%)	4 (42%)	2 (40%)
	70–79	9 (33%)	4 (40%)	3 (25%)	2 (40%)
	80 or above	6 (22%)	1 (10%)	4 (33%)	1 (20%)
Gender	Male	10 (37%)	0 (0%)	6 (50%)	4 (80%)
	Female	17 (63%)	10 (100%)	6 (50%)	1 (20%)
Education	Primary school or lower	4 (15%)	1 (10%)	1 (8%)	2 (40%)
	Junior secondary education	7 (26%)	2 (20%)	4 (33%)	1 (20%)
	Senior secondary education	10 (37%)	5 (50%)	3 (25%)	2 (40%)
	Undergraduate or higher	6 (22%)	2 (20%)	4 (33%)	0 (0%)
Living conditions	Living alone	12 (44%)	5 (50%)	6 (50%)	1 (20%)
	Living with others	15 (56%)	5 (50%)	6 (50%)	4 (80%)
Health conditions	None	2 (7%)	2 (20%)	0 (0%)	0 (0%)
	1 to 2	13 (48%)	4 (40$)	6 (50%)	3 (60%)
	3 or above	12 (44%)	4 (40%)	6 (50%)	2 (40%)
Types of services received	Home cleaning	9 (33%)	3 (30%)	3 (25%)	3 (60%)
	Food delivery	10 (37%)	4 (40%)	5 (42%)	1 (20%)
	Physical therapy/exercises	7 (26%)	1 (10%)	5 (42%)	1 (20%)
	Leisure activities	11 (41%)	6 (60%)	3 (25%)	2 (40%)
	Others (health education, health management)	10 (37%)	4 (40%)	2 (17%)	4 (80%)
Interview mode	Face to face	15 (56%)	8 (80%)	4 (33%)	3 (60%)
	Phone	12 (44%)	2 (20%)	8 (67%)	2 (40%)

**Table 2:** Characteristics of the experts from independent expert review (*N* = 7)

No. of experts	Position	Gender
Expert 1	LTC regulator	Female
Expert 2	Social worker	Male
Expert 3	Social worker	Male
Expert 4	Research nurse	Female
Expert 5	Professional consultant (Nursing)	Male
Expert 6	Research assistant professor	Male
Expert 7	Research assistant professor	Female

### Comprehensibility

During the cognitive interviews, participants generally perceived the instrument as clear, easy to complete, appropriate in length, and reported no sensitive feelings when completing the survey. As shown in Table 3, in the first-round cognitive interviews, five of the nine questions had comprehensibility issues with items, especially ‘*Q3. Food and drink*’ and the two ‘*Q8 & 9. Dignity*’ items, where over half of the participants experienced comprehension and interpretation issues. Four of the nine questions had issues with response options, with around one-third of the participants finding them difficult to understand or distinguish. Most issues were addressed through wording revisions. However, for the ‘*Q3. Food and drink*’ and ‘*Q8 &9. Dignity*’ items, alternative terms were used, and sentence structure adjustments were made to enhance participants’ comprehension. During the independent expert review, all experts rated the instrument as clear, supporting evidence of the comprehensibility of the Chinese ASCOT-SCT4.

**Table 3:** Summary of comprehension and interpretation issues reported by participants of the Chinese ASCOT-SCT4 during cognitive interviews (*N* = 27)

Items	Comprehension and interpretation issues					
	Round 1 (*n* = 10)		Round 2 (*n* = 12)		Round 3 (*n* = 5)	
	Item	Response	Item	Response	Item	Response
Q1. Control over daily life	3	3	-	-	-	-
Q2. Personal cleanliness	3	-	-	-	-	-
Q3. Food and drink	8	3	-	2	-	1
Q4. Personal safety	-	-	-	-	-	-
Q5. Social participation	-	3	-	-	-	-
Q6. Occupation	-	4	-	5	-	-
Q7. Home cleanliness	-	-	-	-	-	-
Q8. Dignity filter	6	-	3	-	1	-
Q9. Dignity	6	-	1	-	1	-

#### Comprehension and interpretation issues—by participants

As shown in Table 4, for the ‘*Q1. Control over daily life*’ item, during the first-round interviews, participants found the term ‘autonomy’ in the Chinese translation difficult to understand. The first response option, intended to convey ‘having as much control as I want’, was often misunderstood as insufficient control and a desire for more. Additionally, participants struggled to distinguish between the first (as much as I want) and the second (adequate) response options, as they perceived the latter as already meeting their desired level of control. This confusion was attributed to the literal translation of ‘adequate (足夠)’ in Chinese, which can imply ‘more than just enough’. To address these, revisions included refining the translation of ‘control’, modifying the first response option, and adding ‘basic’ before ‘control’ in the second response option to convey the intended nuance. Subsequent participants found the revised version comprehensible.

**Table 4:** Detailed illustrations of comprehension and interpretation issues and revisions for the Chinese ASCOT-SCT4 during cognitive interviews (*N* = 27)

Items	Rounds	Findings and identified problems	Illustrative participants’ comments	Revision decisions based on findings
Q1. Control over daily life	1	Odd/difficult wording—Item: 2 participants (2/10) suggested the term ‘autonomy (自主權)’ was not straightforward to understand	- P1: “I can understand this term but it is not quite common in our daily language.” - P3: “When I first read the question, I needed to think about what the meaning of ‘autonomy (自主權)’ is. Although there is an explanation sentence below the question, I hope it could be more straightforward when asking.”	An alternative term, ‘掌控’, was used to replace ‘自主權’.
		Difficult interpretation—Item: 1 participant (1/10) was uncertain about the intended meaning of ‘autonomy (自主權)’	- P9: “I think this term has two meanings: either the person should be active, or the person should do whatever he/she wants based on his/her own preferences. I am not sure which meaning this question refers to.”	
		Wrong interpretation—Response: 3 participants (3/10) misunderstood the meaning of the first response option	- P4: “I think the first option refers to not having enough autonomy and wanting more. If you want me to compare the level of the first and the second options, I would say the second option represents higher level as it means achieving enough. So, for my situation, I chose the first option, because I don’t have enough autonomy, and I want to have more.” - P5: “I think the first option suggests a lack of enough autonomy and wanting more.” - P10: “I think the first option may not achieve sufficient autonomy because it said it is ‘as much as possible’.”	(1) An alternative phrase, ‘與我想要的一樣’, was used to replace ‘想盡可能多’; (2) Following the revision (1), to improve distinction between the first and the second response options, an alternative phrase '基本可以' was used to replace the term '足夠' in the second response option.
	2 & 3	Well comprehended by all the participants (17/17)	/	/
Q2. Personal cleanliness	1	Odd/difficult wording—Item: 2 participants (2/10) thought the wording ‘presentable (體面)’ does not align with their daily language	- P3: “We usually wouldn’t use this term ‘presentable (體面)’ to describe appearance” - P9: “‘得體’ is better than’體面’.”	An alternative term, ‘得體’, was used to replace ‘體面’.
		Difficult interpretation—Item: 2 participants (2/10) did not understand what was meant by ‘presentable (體面)’	- P3: “I don’t know what ‘presentable (體面)’ means. Does it mean that you have to dress up in expensive and beautiful clothes?” - P4: “It’s difficult to interpret the term ‘presentable(體面)’.”	
	2 &3	Well comprehended by all the participants (17/17)	/	/
Q3. Food and drink	1	Difficult interpretation—Item: 4 participants (4/10) were confused about the range of ‘getting food and drink (獲得食物和飲品)’	- P1: “Homemade or outside food?” - P4: “The question is not clear, it does not explain how the food was obtained, whether it was cooked by me or given by others?” - P5: “Does ‘getting food’ refer to buying food from outside?” - P9: “I am thinking about whether it refers to eating outside or homemade food…”	The term ‘getting (獲得)’ was deleted a)nd replaced with a new term ‘daily (日常的)’ in the question sentence.
		Narrow interpretation—Item: 4 participants (4/10) narrowly interpreted the range of this item	- P3, P6, P7, P10: “Getting food refers to someone giving only.”	
		Odd/difficult wording—Response: 1 participant (1/10) thought the wording ‘don’t always (並非時常)’ does not align with their daily language	- P3: “‘不是經常’ sounds better than ‘並非時常’ as the former one is more commonly used in our daily language.”	An alternative term, ‘不是經常’, was used to replace ‘並非時常.’
		Wrong interpretation—Response: 3 participants (3/10) misunderstood the fourth option	- P3: “I will choose the fourth option, when I encounter this situation: for example, I don’t like to eat lunch meat because I think it is not good for me; however, if I travel to countryside, I have no choice but to eat lunch meat, then I think it will be a risk to my health.” - P7: “The last option means the food and drink will negatively influence people’s health.” - P9: “People don’t always get adequate or timely food and drink because those food and drink can negatively influence their health. For example, people who have diabetes or hypertension will have limitations in dietary intake.”	The sentence was restructured: ‘and I think there is a risk to my health (我認為這樣會危害我的健康)’ was replaced with ‘which harms my health (而危害我的健康)’.
	2	Wrong interpretation—Response: 2 participants (2/12) misunderstood the fourth option	- P11: “I think no one will choose the fourth option as no one would eat food to harm their health…” - P21: “The last option can be interpreted as: I don’t always eat food that is hazardous to my health. For example, drinking sodas and eating something unhealthy will harm my health.”	Retained and retested.
	3	Wrong interpretation—Response: 1 participant (1/5) misunderstood the fourth option	- P23: “Probably the food has high salt and sugar, so not always get it?”	Retained.
Q4. Personal safety	1 & 2 & 3	Well comprehended by all the participants (27/27)	/	/
Q5. Social participation	1	Odd/difficult wording—Response: 1 participant (1/10) did not understand the intended meaning of the first option	- P5: “when I first read the first response option, I just literally skipped it because I thought it doesn’t read smoothly and requires some time to understand.”	An alternative phrase, ‘與我想要的一樣’, was used to replace ‘想盡可能多’.
		Wrong interpretation—Response: 2 participants (2/10) misunderstood the first option	- P1: “The first option refers to more than enough social contact. But I already have enough social contact; it sounds very tiring to have more than enough.” - P10: “The first option means my social contact is not enough, and I want more.”	
	2 & 3	Well comprehended by all the participants (17/17)	/	/
Q6. Occupation	1	Odd/difficult wording—Response: 2 participants (2/10) thought the wording ‘as I want (隨心所欲)’ is impossible to achieve	- P5: “This term is too ideal to achieve.” - P6: “‘隨心所欲’ is good, but it is impossible.”	An alternative phrase, ‘隨心’ was used to replace ‘隨心所欲’.
		Difficult interpretation—Response: 2 participants (2/10) found it difficult to differentiate between the first and the second options	- P4: “What’s the difference between these two? The first one is to do whatever you want, the second one is to fully…emm (then thinking for a long time). Maybe the first option refers to no planning in doing things, and the second option refers to having planning?” - P8: “I can’t get the difference between the first and the second option.”	An alternative phrase, ‘基本可以’ was used to replace ‘充分地’.
	2	Difficult interpretation—Response: 5 participants (5/12) found it difficult to differentiate the first and the second options	- P13, P14, P15, P21, P22: “The first and the second option look very similar.”	
	3	Well comprehended by all the participants (5/5)	/	/
Q7. Home cleanliness	1 & 2 & 3	Well comprehended by all the participants (27/27)	/	/
Q8. Dignity filter	1	Odd/difficult wording—Item: 2 participants (2/10) struggled to understand the sentence	- P3: “The sentence looks unusual and odd; I think it’s hard to understand.” - P8: “I didn't quite understanding it at first glance.”	The term ‘dignity (自尊)’ was used to replace the phrase ‘think and feel about myself (對我的看法和感覺)’.
		Difficult interpretation—Item: 2 participants (2/10) did not understand the intended meaning of this question	- P7: “What does it mean to think and feel about oneself? I think the clarity is 80% only, because I don’t quite get the meaning of it.” - P10: “I don’t understand why someone helping you would change your thoughts?”	
		Wrong interpretation—Item: 3 participants (3/10) misunderstood the intended meaning of the question	- P1: “Everyone has a different opinion. If you think that the person offering help may be right after he/she has spoken, you will refer to his/her opinion, but you may not necessarily accept it. So, (I feel that) person who offers help will (or could) undermine my views and feelings.” - P3: “For example, if I go to take a bus and someone keeps telling me not to take the bus but to take a car or the MTR or something like that, it will affect my view.” - P6: “After receiving bathing help, I changed my view on the quality of service as I found the helpers were quick to help with the shower because they were in a hurry to get off work. I feel disappointed with the service.”	
	2	Difficult interpretation—Item: 2 participants (2/12) found it hard to interpret the intended meaning of the question	- P13: “I do not quite understand this question.” - P17: “It’s difficult to analyze this question. How would it affect my self-dignity? Can you give me some examples?”	Retained and retested.
		Broad interpretation—Item: 1 participant (1/12) said it would influence self-confidence	- P21: “I am a person who lacks confidence. If someone can help me, it will greatly improve my self-confidence.”	
	3	Broad interpretation—Item: 1 participant (1/5) said it would influence self-confidence	- P27: “It (having help) affects my self-confidence.”	The term ‘self-confidence (自信)’ was added to the question.
Q9. Dignity	1	Difficult interpretation—Item: 1 participant (1/10) did not understand what was meant by ‘the way of being helped (方式)’	- P10: “I don’t understand the way of being helped (對待方式).”	(1) An explanation sentence of the way of being helped was added to the question; (2) The term ‘self-esteem (自尊)’ was used to replace the phrase ‘think and feel about myself (對我的看法和感覺)’.
		Wrong interpretation—Item: 5 participants (5/10) did not distinguish the differences between Q8 and Q9	- P2, P3, P6, P7, P8 interpreted this question the same as Q8, did not mention the way of being helped.	
	2	Broad interpretation—Item: 1 participant (1/12) said it would influence self-confidence	- P21: “Attitude is important. My daughter taught me how to use mobile phone with patience, unlike my son. So, I think the way of being helped has improved my confidence and dignity.”	The term ‘self-confidence (自信)’ was added to the question.
	3	Broad interpretation—Item: 1 participant (1/5) said it would influence self-confidence	- P27: “It (the way of being helped and treated) won’t affect my self-dignity but would affect my self-confidence.”	

Regarding the ‘*Q3. Food and drink*’ item, most participants were confused about whether ‘getting food and drink’ included food and drink they prepared themselves or referred exclusively to provisions from others. To resolve this, ‘getting’ was replaced with ‘daily’, and no confusion was reported in subsequent rounds. Misinterpretation of the fourth response option was observed, with some participants interpreting it to mean that individuals do not receive adequate or timely food and drink because these items could negatively impact their health. This issue arose because the term ‘there’ was interpreted as referring to ‘food and drink’ rather than the overall sentence. Removing the term ‘there’ and restructuring the sentence improved comprehension in subsequent interviews. However, three out of seventeen participants in later rounds still misinterpreted this response option. Upon clarification by interviewers, they understood the intended meaning but noted that such a situation is uncommon in HK, particularly among community-dwelling individuals. As this appeared to stem from personal experiences rather than translation issues, the translation was retained to align with the source version.

For the ‘*Q5. Social participation’* item, the literal translation of the first response option led to misinterpretations, with some participants perceiving it as insufficient social contact. Revising the wording resolved this issue and clarified the intended meaning.

For the ‘*Q6. Occupation’* item, around one-third of the participants across the first two interview rounds struggled to distinguish between the first two response options because the literal translation of ‘enough’ did not convey the intended meaning. The second response option was revised to ‘I’m basically able to…’, and participants in subsequent rounds found the distinction clear.

For the two ‘*Q8 & 9. Dignity*’ items, significant issues with interpretation and comprehension were identified in the first interview round. Two participants found the items unclear, two struggled to relate receiving help to self-perception, and three misunderstood the questions. Some participants could only understand the questions’ meaning when prompted with terms such as ‘self-esteem’ and ‘self-confidence’. Consequently, ‘think and feel about myself’ was replaced with ‘self-esteem’ in both items. Additionally, around half of the participants found it difficult to distinguish between the two ‘*Dignity*’ items, and one participant questioned which types of help should be considered. To clarify, a standard example was added to explain that ‘the way of being helped and treated’ in the ‘*Q9. Dignity*’ question refers to attitudes and types of help (encouragement/material/invisible help). These revisions improved comprehension in subsequent rounds, though some participants still required multiple readings. As some participants mentioned that having help would influence their ‘self-confidence’ rather than ‘self-esteem’, the term ‘self-confidence’ was included in the questions as well.

#### Clarity—by experts

Table 5 shows that the I-CVIs for individual items ranged from 0.86 to 1.00, and the S-CVI for the entire pre-final instrument was 0.92, demonstrating excellent clarity. Among the seven experts, one recommended changing the wording of the fourth response option in the ‘*Q3. Food and drink*’ item to ‘I am not able to get…’. Another expert suggested adding “but it is not what I want/like” to the second response option to clearly differentiate it from the first response option in ‘*Q2. Personal cleanliness*’ and ‘*Q7. Home cleanliness*’ items. Additionally, this expert noted the distinction between the ‘*Q8. Dignity filter*’ and ‘*Q9. Dignity*’ questions was not clear due to similar wording and overlapping concepts, recommending the removal of the ‘*Q8. Dignity filter*’ to avoid participant confusion. As these suggestions diverge significantly from the source version, our research team decided not to further revise the instrument to maintain the equivalence between the Chinese and English versions. The final version of the Chinese ASCOT-SCT4 was approved by the UK developers.

**Table 5:** Ratings on clarity of the Chinese ASCOT-SCT4 by experts from the independent expert review (*N* = 7)

Items	Expert agreement	I-CVI^a^	Comments^c^	Decisions
Q1. Control over daily life	7/7	1.00	/	
Q2. Personal cleanliness	6/7	0.86	One expert (1/6, E7) suggested adding “but it may not be what I want/like” to the second option.	Retained
Q3. Food and drink	6/7	0.86	One expert (1/6, E6) suggested revising the fourth option to “I am not able to get enough or timely food and drink, which harms my health”.	Retained
Q4. Personal safety	7/7	1.00	/	/
Q5. Social participation	7/7	1.00	/	/
Q6. Occupation	7/7	1.00	/	/
Q7. Home cleanliness	6/7	0.86	One expert (1/6, E7) suggested adding “but it may not be what I want/like” to the second option.	Retained
Q8. Dignity filter	6/7	0.86	One expert (1/6, E7) questioned the difference between the ‘*Dignity filter*’ and ‘*Dignity*’ questions and suggested removing the ‘*Dignity filter*’ item.	Retained
Q9. Dignity	6/7	0.86		
S-CVI/Ave^b^		0.92		

^a^I-CVI, item-level content validity index; I-CVI is calculated as the number of experts giving ratings on clarity of either 3 (quite clear) or 4 (very clear) divided by the total number of experts

^b^S-CVI/Ave, scale-level content validity index; S-CVI/Ave is calculated as the average of I-CVIs for all the nine items

^c^Comments were provided only for expert ratings of 1 (Not clear) or 2 (somewhat clear)

### Relevance

Participants and experts generally reported that the eight domains were relevant for LTC outcomes in the HK context. As shown in Table 6, the S-CVI/Ave for the overall instrument was 0.97, and the I-CVI across the eight domains ranged from 0.94 to 1.00, indicating the instrument is highly relevant at both the scale and item levels.

**Table 6:** Ratings on relevance of the Chinese ASCOT-SCT4 by participants from cognitive interviews and experts from the independent expert review

Items	Total (*N* = 34)		Participants (*n* = 27)		Experts (*n* = 7)	
	**Agreement**	**I-CVI**^**a**^	**Agreement**	**I-CVI**^**a**^	**Agreement**	**I-CVI**^**a**^
Q1. Control over daily life	34/34	1.00	27/27	1.00	7/7	1.00
Q2. Personal cleanliness	34/34	1.00	27/27	1.00	7/7	1.00
Q3. Food and drink	32/34	0.94	25/27	0.93	7/7	1.00
Q4. Personal safety	33/34	0.97	26/27	0.96	7/7	1.00
Q5. Social participation	33/34	0.97	26/27	0.96	7/7	1.00
Q6. Occupation	33/34	0.97	26/27	0.96	7/7	1.00
Q7. Home cleanliness	33/34	0.97	26/27	0.96	7/7	1.00
Q9. Dignity	32/34	0.94	25/27	0.93	7/7	1.00
S-CVI/Ave^b^		0.97		0.96		1.00

^a^I-CVI, item-level content validity index; For each group in this table (Total, Participants, Experts), the I-CVI is calculated as the number of raters giving ratings on relevance of either 3 (quite relevant) or 4 (very relevant) divided by the total number of raters in that specific group

^b^S-CVI/Ave, scale-level content validity index; S-CVI/Ave is calculated as the average of I-CVIs for all the eight domains

### Comprehensiveness

Ten of the 27 participants mentioned the absence of health-related domains such as vitality, sleep quality, and mental health, which they considered important to their QoL. However, as health is not the primary focus of LTC services and the instrument is designed to evaluate LTC-related QoL, these health-related domains were not considered key attributes of the instrument. When participants were asked to propose additional domains beyond health, they did not suggest any others. One participant recommended including a domain related to the accessibility of care and support services. However, accessibility is generally not considered an outcome of LTC but rather a system-level determinant influencing the achievement of outcomes. Furthermore, no additional key domains were recommended by the experts, supporting the comprehensiveness of the instrument within its intended scope.

## Discussion

This study involved the translation, cultural adaptation, and content validation of the ASCOT-SCT4 instrument for use with LTC users in HK. Cognitive interviews revealed that literal translations of most items and responses led to misunderstandings and difficulties in interpretation—particularly for ‘*Food and drink*’ and ‘*Dignity*’ items, as well as the first two response options related to ‘*Control over daily life*’, ‘*Social participation*’ and ‘*Occupation*’, underscoring the critical need for cross-cultural adaptation to ensure the instrument is appropriate for the local context. Rewording and rephrasing were employed to improve comprehension in items and clarify distinctions between response options. Both scale- and item-level CVIs were satisfactory, confirming that the Chinese ASCOT-SCT4 is clear and relevant for use in HK. No additional LTC-related QoL domains were identified as missing from the instrument by participants or experts. Given the limited existing evidence of the content validity of the ASCOT-SCT4 [42, 44], our study demonstrates that the instrument is comprehensible, relevant, and comprehensive in the HK context. These findings also pave the way for further testing of its psychometric properties in a larger Chinese population.

Due to the linguistic and cultural differences between Chinese and English, we encountered several challenges in which literal translations of certain terms were either incomprehensible or diverged from the intended meaning. These issues were particularly evident with the term ‘control’ in the ‘*Control over daily life*’ item, the term ‘getting’ in the ‘*Food and drink*’ item, and the phrase ‘(having helped or) the way of being helped and treated affects the way I think and feel about myself*’* in the ‘*Dignity*’ items. These challenges align with findings from previous cross-cultural adaptation studies of the ASCOT-SCT4 [19, 21, 22]. The Dutch [19], Austrian [21], and Japanese [22] teams considered the term ‘control’ too rigid, as it evoked feelings of being scrutinized, and replaced it with terms like ‘arrange [19]’, ‘self-determined [21]’ or ‘decide by yourself [22]’. The Dutch study found that ‘get’ in the ‘*Food and drink*’ item was ambiguous, as it was unclear whether individuals obtained food and drink themselves or received them from others; therefore, the translation was revised to ‘can eat and drink’ [19]. Regarding the ‘*Dignity*’ items, participants in the Dutch and Austrian studies were confused by the relationship between being helped and its impact on their thoughts and feelings; as a result, alternative terms such as ‘treated with respect [19]’, ‘self-image [19]’, or ‘self-esteem [21]’ were used.

In the current study, the literal translation of the term ‘control (控制)’ in Chinese may imply a rigid or authoritative sense. To avoid evoking sensitive feelings and with reference to previous studies, we initially considered replacing ‘control’ with ‘autonomy’ or ‘self-determined’. However, cognitive interviews revealed that some participants made a distinction between decision-making and the ability to act on decisions, which affects how they respond to the item, noting that while they could make decisions independently as they lived alone, their health conditions often limited their ability to act on these decisions. Therefore, we retained ‘control’ but replaced its literal translation with a more neutral term (‘掌控’), which we believe is semantically and conceptually equivalent to the source version. However, this raises concerns about cross-cultural differences and potential systematic variations in responses across countries/regions due to the use of different terms in various language versions. A similar concern arose with the ‘*Dignity*’ item. The phrase ‘think and feel about myself’ in the English version [17] was an uncommon expression in HK and other countries [19, 21]. In our study, ‘self-esteem’ and ‘self-confidence’ were adopted, as these terms are more familiar in everyday language in HK; however, they differ from terms used in English [17], Dutch [19], and Japanese [22] versions. The cross-cultural adaptation of the items highlights the need for further research on measurement equivalence across different language versions (i.e., by conducting differential item functioning analyses [45]).

The ASCOT connects capability theory to reflect the notion of freedom of choice through its first response option, while the remaining three response options indicate functioning states [17]. This innovative design aims to capture whether LTC services are provided according to individual preferences; however, it may reduce the simplicity and clarity of response levels, thereby increasing cognitive burden and causing misinterpretation [46, 47]. In our study, participants struggled to differentiate between the first two response options in the ‘*Control over daily life*’ and ‘*Occupation*’ items, with some even perceiving the second as representing a higher level than the first. This could be partly explained by cultural differences, reflected in the literal translation of ‘adequate/enough’ in Chinese, which may imply that needs are fully met or even exceeded, potentially overlapping with the notion of personalization. In Chinese culture, softer phrases like ‘basically enough’ are commonly used to humbly express that something is just sufficient to meet needs, which better aligns with the intended meaning of the second response option. The addition of the term ‘basic’ improved the distinction; however, the differences between the first two response options remain subtle, as noted by one of the experts in our study and in previous content validation studies conducted among Dutch and Australian populations [42, 48]. Since this issue originates from the design of the English version, the phrasing and structure of the response options were retained to ensure cross-cultural equivalence. Nevertheless, the limited distinction may introduce response bias, which could compromise test–retest reliability. This warrants further investigation, and if the issue persists, modifications to the structure or alternative phrasing should be considered.

The eight domains in the ASCOT-SCT4 were selected for their significant impact on individuals’ QoL and the critical role that social care services can play, using a top-down approach through literature review and expert views [17, 49]. As the ASCOT-SCT4 was developed in the UK, the relevance of its domains may vary across cultural contexts due to differences in social care systems and cultural perceptions of QoL. Additionally, perspectives from target populations may differ from those of experts, highlighting the need to evaluate relevance from both viewpoints [50]. Our study demonstrated the relevance of these domains for assessing outcomes of LTC in the HK context, as indicated by high CVIs agreed upon by both LTC users and experts.

Consistent with previous studies, this study found that health was frequently mentioned by participants as one of the important aspects of their QoL [17, 49]. However, the absence of health-related domains in the instrument should not be considered a lack of comprehensiveness. According to the conceptual basis of the ASCOT, health was not included as it is not the primary focus of LTC which aims to compensate for individuals who experience a loss of functional ability rather than directly improve their health [17, 49]. Focusing on outcomes specific to LTC ensures the instrument sensitively captures its impacts and aligns with the priorities of decision-makers who allocate resources to maximize the effectiveness of LTC services [17, 51]. Given the importance of health to individuals’ overall QoL and the potential influence of LTC on health, using health-related QoL instruments, such as EQ-5D, alongside the ASCOT is recommended to capture both health and non-health benefits of LTC interventions [12, 23, 52]. Nonetheless, using both instruments simultaneously brings attention to potential overlap in the constructs they measure, which could lead to double counting benefits or challenges in interpreting results. Future research should explore the relationship between LTC-related QoL and the health-related QoL to clarify potential overlaps and ensure that evaluations provide reliable, non-redundant, and comprehensive insights into the benefits of LTC services.

This study has several limitations. Firstly, although we recruited a relatively large sample of care service users to test and adapt the instrument, all participants were from community care settings. This may limit the generalizability of our findings to residential care settings, where recipients may have different levels of understanding and interpretation. Secondly, due to COVID-19 restrictions and safety concerns, some cognitive interviews were conducted over the phone rather than face-to-face. This, however, may introduce differences in the question-answering process. For example, nonverbal cues such as facial expressions could not be observed. To minimize these potential effects, we followed the same interview procedure and guideline across both modes, with all interviews conducted by the same two well-trained interviewers. During phone interviews, verbal cues such as pauses or hesitations were closely monitored, and follow-up questions were asked to ensure comprehension. In the present study, most of the comprehension and interpretation issues were identified during the first-round cognitive interviews, where 80% of the interviews were conducted face-to-face. The instrument was subsequently revised and tested in the later rounds, during which a significant reduction in issues raised by participants was found. This suggests the revised instrument was comprehensible regardless of the interview mode. Nevertheless, we cannot entirely rule out the possibility of mode-related differences that may have influenced the results. Thirdly, the absence of male participants in the first round may have missed gender-specific feedback. However, the overall gender distribution (37% male, 63% female) aligns with the latest census statistics on older adults with LTC needs [53] (used as a proxy for the LTC users due to the lack of public statistics on the target population), suggesting the study sample reasonably reflects the target population in terms of gender. Lastly, to reduce interview burden, comprehensiveness was evaluated by asking participants whether any domains important to their QoL were missing from the instrument. While this approach has been used in previous content validity studies [42, 54], it may restrict participants’ reflections to the instrument’s predefined framework, potentially overlooking crucial aspects of their QoL. Further research could adopt a more comprehensive method, such as by asking participants to describe what they consider important to their QoL before reviewing the questionnaire, then mapping these aspects to the instrument to evaluate whether it adequately covers all relevant domains.

## Conclusion

This study demonstrated the translation and cross-cultural adaptation process of the ASCOT-SCT4, originally developed in the UK, for use with LTC users in HK. Semantic and conceptual equivalence were established by collaboration with the UK developer team and a professional translation company, and were further supported by LTC users and experts. Both qualitative and quantitative evidence confirmed that the Chinese ASCOT-SCT4 is comprehensible, relevant, and comprehensive in the HK context. In further research, we plan to validate the psychometric properties of the instrument to assess the QoL of LTC service users. A valid tool enables policymakers and service providers to monitor the quality of LTC over time, ensure services meet recipients’ needs, evaluate their impact on the QoL outcomes, and aid (cost-)efficient resource-allocation.

## Supplementary Information

Supplementary Material 1.

## Data Availability

The data that support the findings of the current study are available from the corresponding authors on reasonable request.
