# Supplementary material for: Translation, cultural adaptation, and content validation of the Hong Kong Chinese version of Self-completion Adult Social Care Outcomes Toolkit (ASCOT-SCT4) for care service users
Source: Health Qual Life Outcomes. 2025 Jun 4;23:57. doi: 10.1186/s12955-025-02389-5 (PMC12135604; doi:10.1186/s12955-025-02389-5)
Supplement: Supplementary file 1 — Supplementary Material 1. [file 12955_2025_2389_MOESM1_ESM.docx]

**Supplementary files**

Contents

[Table S1. COSMIN reporting guideline for studies of measurement properties 2](#_Toc187098599)

[Table S2. Domain and definitions of the ASCOT-SCT4 7](#_Toc187098600)

[Table S3. Modified coding framework 8](#_Toc187098601)

[Interview guideline 9](#_Toc187098602)

# Table S1. COSMIN reporting guideline for studies of measurement properties

| General reporting recommendations relevant for all studies on measurement properties | | | |
| --- | --- | --- | --- |
| Item number | Item name | Item description | Location where item is reported |
| Report section: title | | | |
| T1 | Patient-reported outcome measure (PROM) | The name of the PROM instrument(s) (and version if relevant) being studied | Page 1 |
| T2 | Measurement property (MP) | What MPs are being studied or more generally, that MPs are being studied (if there are many properties being investigated, for example) | Page 1 |
| T3 | Study sample | General description of relevant study sample characteristics (e.g., condition of interest, language) and also any intervention or exposure (e.g., treatments) if applicable | Page 1 |
| Report section: abstract | | | |
| A1 | PROM | The name of the PROM instrument(s) (and version if relevant) being studied (i.e., the SF-36 or SF-12; language version) or if it concerns an item bank (e.g., PROMIS instruments). The type of instrument (e.g., a self-reported questionnaire or interview) | Page 2 |
| A2 | Measurement property | What MPs are being studied or more generally, that MPs are being studied (if there are many properties being investigated, for example) | Page 2 |
| A3 | Design | The type of study being used to test the properties (e.g., test–retest design, longitudinal study, cohort, cross sectional, case series, randomized etc.). Other details of the study design if relevant (intervention/exposure, description of comparison instruments, outcomes other than PROMs) | Page 2 |
| A4 | Sample | Inclusion / exclusion criteria. General description of relevant study sample characteristics (e.g., condition of interest, geographic location, language, other relevant demographic and baseline characteristics) | Page 2 |
| A5 | Methods | A brief description of the methods for investigating each MP including statistical analyses | Page 2 |
| A6 | Results | The main results for all MPs investigated reporting statistics for each result with measures of precision where appropriate | Page 2-3 |
| A7 | Discussion/Conclusions | A brief description of the results in the context of existing evidence, main strengths and drawbacks and the need for future research on the PROM(s) investigated | Page 3 |
| Report section: introduction | | | |
| I1 | Name and describe the PROM of interest | Specify the name, type, language, and version of the PROM being investigated and how it was developed. Describe the construct the PROM aims to measure and its subscales; describe the structure of the PROM (e.g., the number of factors, the number of items, scoring algorithm); describe relevant instructions (like time period), and number or type of response categories. State whether the PROM is based on a reflective or formative model  Note: This information may also appear in the methods section in greater detail | Page 4-5 |
| I2 | Target population | Describe the specific target population that the PROM was designed for. The authors need to provide the appropriate and necessary characteristics of this population | Page 4-5 |
| I3 | Citation for the original development of the PROM | The citation for the original development paper(s) should be provided and other highly relevant citations related to the quality of the specific PROM under investigation | Page 4 |
| I4 | State of knowledge & Rationale | A description of the current scientific knowledge (what is known) regarding the MPs of? the PROM under investigation. The authors should provide a literature review or refer to a recent review of all existing evidence of the specific version (e.g., language, short form) of the PROM and explain why the new study is necessary and important. The rational for the current proposed study should be given | Page 4-5 |
| I5 | Definitions | Specialized terms should be defined or explained | Page 5-6 |
| I6 | Objectives and hypotheses | State the specific objective(s) of the research and hypotheses related to the specific PROM under investigation | Page 5-6 |
| Report section: general methods | | | |
| GM1 | Study design | State the key elements of the study design | Page 6-9; Figure 1 |
| GM2 | Participants | State how the participants were chosen; the inclusion and exclusion criteria. (e.g., if a PROM for a specific condition, then the eligibility and selection criteria should reflect this) | Page 7-8, Page 9-10 |
| GM3 | PROM administration | An explicit description of how and when the PROM(s) were administered (e.g., in what setting) including data collection devices/system used (e.g., paper-based, electronic administration / ePRO) should be provided | Page 9-10 |
| GM4 | Data collection procedures | Provide information about other data collection, exposure methods (e.g., allocation to interventions) and time points / follow-up points | Page 7-10; Page 11 |
| GM5 | Power/sample size calculation | Provide a power calculation for all MP analyses. Alternatively, if a rule of thumb is used, state it and the source/citation | Page 8, Page 9 |
| GM6 | Statistical analyses | Statistical analyses and tests corresponding to all hypotheses or objectives for all MPs should be reported. Where appropriate, a cutoff for statistical significance should be reported (e.g., p-value less than 0.05). A description of all statistics to be used to estimate the magnitude and direction of effect should also be reported, together with measures of variability or precision. Report statistical package used | Page 10 |
| GM7 | Missing data | State approaches or plan for dealing with missing data | NA |
| GM8 | Post hoc analysis | The report should specify analyses that used data after the data collection period concluded (i.e., if the analyses were post hoc; secondary data analyses) and describe the rationale for any post hoc analyses | NA |
| Report section: general results | | | |
| GR1 | Missing data | The amount and reasons for missing data should be explained for all analyses for all PROMs (or other outcome measurement instruments) and relevant groups | NA |
| GR2 | Participant/patient Characteristics | The study patients’ characteristics should be described, including baseline PROM scores | Page 11 |
| GR3 | Sample size | If one study contained analyses using different sample sizes, the authors should report the sample size for each analysis | Page 11; Table 3, 5, 6 |
| Report section: discussion | | | |
| D1 | MP evidence | Per measurement property the authors should compare the result to the criteria for good measurement properties (e.g., COSMIN criteria) [[25](https://link.springer.com/article/10.1007/s11136-021-02822-4#ref-CR25)], and determine if the specific MP is sufficient or not. Note: This information may also appear in the results section in greater detail in a table for example | Page 14-15 |
| D2 | Practical relevance | The authors need to discuss the practical relevance of the findings | Page 21 |
| D3 | Strengths and limitations | Strengths and limitations of the study should be discussed. For example, discuss if there were any significant potential biases in the study that could have impacted the results | Page 20-21 |
| D4 | Generalizability | Generalizability issues related to the PROM results should be discussed. For example, discuss if the results could be generalized to other populations given the sample studied | Page 20 |
| D5 | Instrument changes | Discuss the need for modifications to the existing PROM or new PROM development. If you conclude that one of the measurement properties is insufficient, you could suggest some modification, or if it is really poor, you could suggest stopping use of the PROM (in the specific population or in general) | Page 18 |
| D6 | Future research | Report specifically the type of research needed to answer new questions arising out of these findings for the particular MP and PROM investigated | Page 17-21 |
| Report section: conclusions | | | |
| C1 | Conclusions | State the overall conclusions for each MP and of the use PROM investigated | Page 21 |
| Report section: other information | | | |
| O1 | Conflict of interest | State any relevant conflict of interest related to the PROM under investigation (e.g., an author being the PROM developer, funding body etc.) | Page 22 |
| Specific reporting recommendations for studies on content validity | | | |
| Item number | Item name | Item description |  |
| CV1 | Relevance | Report if and how patients and/or professionals were asked whether each item is relevant for their experience with the condition | Page 8-10 |
| CV2 | Comprehensiveness | Report if and how patients and/or professionals were asked whether all key concepts are included | Page 8-10 |
| CV3 | Comprehensibility | Report if and how the comprehensibility of the PROM instructions, items, response options, and recall period was assessed | Page 8-10; Interview guideline |
| CV4 | Relevance results | Report if all items were considered relevant for the construct, population, and context of use of interest by patients and/or professionals | Page 15, Table 6 |
| CV5 | Response options and recall period | Report whether the response options and recall period were considered appropriate by patients and/or professionals | Page 11-15, Table 3-5 |
| CV6 | Comprehensiveness results | Report whether patients and/or professionals considered all key concepts to be included in the PROM | Page 15 |
| CV7 | Comprehensibility results | Report whether patients understood the PROM instructions, items, and response options as intended | Page 11-14, Table 3-4 |

# Table S2. Domain and definitions of the ASCOT-SCT4

| **Domains** | **Definitions** |
| --- | --- |
| Control over daily life | The service user can choose what to do and when to do it, having control over his/her daily life and activities |
| Personal cleanliness | The service user feels clean, comfortable, and presentable, or, at best, is dressed and groomed according to his/her personal preferences |
| Food and drink | The service user feels he/she has a nutritious, varied, and culturally appropriate diet with enough food and drink that he/she enjoy at regular and timely intervals |
| Personal safety | The service user feels safe and secure which means that being free from the fear of abuse, falls, physical harm, attacks, or robbery |
| Social participation | The service user is satisfied with their social situation, which means maintaining meaningful relationships with friends and family and feeling involved or part of a community should this is important to him/her |
| Occupation | The service user is sufficiently occupied in a variety of meaningful activities, including formal employment, unpaid work, caregiving, or leisure activities |
| Home cleanliness | The service user feels that his/her home environment, including all the rooms, is clean and comfortable |
| Dignity | The negative and positive psychological impact of support and care on the service user’s personal sense of significance |

Reference source: Netten A, Burge P, Malley J, Potoglou D, Towers A-M, Brazier J, et al. Outcomes of social care for adults: developing a preference-weighted measure. Health technology assessment. 2012;16:1–166.

# Table S3. Modified coding framework

| **Response issues** | **Categories** | **Definitions** |
| --- | --- | --- |
| Comprehension |  |  |
|  | Odd/difficult wording | Respondents perceive the wordings/phrases are unusual or difficult to understand |
| Interpretation |  |  |
|  | Difficult interpretation | Respondents have difficulty interpreting the meaning of the item/responses |
|  | Wrong interpretation | Respondents interpret the items/responses differently from the intended meaning |
|  | Narrow interpretation | Respondents interpret a narrower range of the construct than what the developers intended to cover |
|  | Broad interpretation^a^ | Respondents interpret a broader range of the construct than what the developers intended to cover |

Reference source: Karen M van Leeuwen

a. ‘Broad interpretation’ was included to the coding framework developed by Karen to categorize our qualitative data.

# **Interview guideline**

**Introduction**

***Explanation of the aim of the study***

- Thank you for participating in this interview. Our research team is developing an instrument to measure the quality of life of care service users. This instrument is called ASCOT, which originated in the UK. The aim of this study is to evaluate whether the Chinese-translated version of this instrument is clear, understandable, relevant to your quality of life, and appropriate for use in the Hong Kong context.

***Explanation of the procedure of the interview***

- First, we will ask you to complete the survey. Once you’ve completed it, we’ll ask you a series of questions about your understanding of the items, as well as why you made the choices you did. The interview is expected to take approximately 30-60 minutes.

***Ethics Declaration***

- This interview will be audio-recorded. Please be assured that your personal information will remain confidential. The information you provide will only be used for research purposes.
- Your participation is voluntary, and you can withdraw from the interview at any time if you feel uncomfortable or for any other reason, without any consequences.

**Main part**

Now, let’s begin the interview. Please start by completing the instrument. You are encouraged to speak aloud your thoughts as you proceed through the instrument. This will help us understand your thought process and how you interpret the questions.

***Part I: General impression***

1. Overall, do you think this questionnaire is clear and easy to complete? Were there any parts that you found confusing or difficult to understand?
2. How do you feel about the length of the questionnaire? Would you consider it too short, appropriate, or too long for you?

***Part II: Comprehensibility***

Now, let’s go through the instrument items and response options one by one.

1. What do you think the meaning of the item and its response options is? Can you explain them in your own words? Could you provide an example to explain it?
2. Are you able to distinguish between the different response options? Can you explain the difference between them?
3. Do you think this question and its response options are clear and easy to understand? Are there any words or phrases that seem unclear or unusual to you?

- Follow-up question: If you find any part unclear or difficult to comprehend, could you suggest revisions to improve its clarity and understandability?

1. You selected [response option]. Can you explain why you chose this option?

- Follow-up question: why you don’t choose the other response options?

***Part III: Relevance***

1. Do you think the items in this instrument are relevant to your quality of life? Specifically, when you are receiving care services, do you feel that the domains (such as control over daily life) are important? How do these domains affect your quality of life?
2. On a scale of 1 to 4 (with 1 being irrelevant and 4 being very relevant), how would you rate the relevance of the items to your quality of life?

***Part IV: Comprehensiveness***

1. This instrument is designed to include key domains that care and support services can deliver or influence and that are important to care receivers’ quality of life. Do you think there are any domains important to your quality of life, which could be influenced or delivered by LTC, but are not covered by this instrument?

***Part V: Others***

1. Did you feel uncomfortable or sensitive about any of the questions while completing the survey?
2. Do you have any questions or concerns about the instrument that you'd like to ask?"

**Close**

Thank you once again for your participation. Your feedback is valuable in helping us refine the instrument and assess its suitability for use in the Hong Kong context.
